# What factors influence the likelihood of rural farmer participation in digital agricultural services? experience from smallholder digitalization in Northern Ghana

**DOI:** 10.1177/00307270221144641

**Published:** 2022-12-26

**Authors:** Abdul-Rahim Abdulai, Krishna Bahadur KC, Evan Fraser

**Affiliations:** 1 Department of Geography, Environment and Geomatics, 3653 University of Guelph, Guelph, Ontario, Canada; 2 Arrell Food Institute at the University of Guelph, 3653 University of Guelph, Guelph, Ontario, Canada

**Keywords:** Digitalization, rural smallholders, Africa, digital participation, inclusive digitalization

## Abstract

Participation in digital services is critical for the inclusiveness of digitalization in smallholder Africa. However, farmers engagement with digitalization services needs further explorations due to limited empirical research on the topic. This paper thus employs a cross-sectional survey of 1565 farmers in Northern Ghana to assess the factors that affect the likelihood of farmers’ participation in digital agricultural services. We applied a polynomial regression model to show that gender, affiliations to farmer groups, access to extension services, ability to place phone calls, and ownership/access to mobile phones increase the probability of participation in digital services. Thus, farmer characteristics, digital competencies, and access to digital resources are critical in determining who participates in digitalization, essentially positioning these as critical factors to consider in scaling of digital agriculture services. We further argue that access and impacts of digitalization could be exclusive due to existing equities in the identified fundamental elements for participation, adoption, and use of digitalization. *Hence, strategies sensitive to the drivers of engagement, including strengthening farmer associations/groups, increasing access to extension services, building digital skills, and scaling access to digital tools (including mobile phones), are required for inclusiveness, scaling and the long-term sustainability of digitalization for smallholders*.

## 1. Introduction

As digital agricultural services are increasingly promoted for smallholder Africa (Kim et al., 2020; Tsan et al., 2019), their adoption and use must be well-understood to inform scaling in the region. Particularly, assessing the participation in digitalization services could propel progress toward solving the challenges that hinder Africa's 33 million smallholders from attaining their productivity and economic potentials (Deutsche Gesellschaft für Internationale Zusammenarbeit et al., 2021; Kim et al., 2020; Technical Centre for Agricultural and Rural Cooperation, 2019). Likewise, however transformative digitalization's potential may be proclaimed in existing literature, attaining such goals partly depend on the beneficiaries’ ability to take advantage of emerging digital services. Yet, the literature on digitalization in Africa remains thin on the factors that may influence the adoption and participation in digital services for diverse groups of rural farmers (FAO, 2019). Hence, a substantial issue in understanding emerging digital agriculture continues to be the limited empirical and systematic data on the topic across different regions and places (ibid). This paper contributes to closing this important gap by empirically modelling the effects a range of factors may have on farmers’ participation in digital agricultural services in Northern Ghana. Specifically, we determines the key factors that influence farmers’ participation in digital agricultural services in the region. We focus on what current characteristics of rural farmers can reveal about the effects different variables may have on the likelihood of engaging with available digital services.

Rural farmers’ existing conditions and characteristics are critical to their participation in interventions (Abegunde et al., 2020; Ali, 2012; Sulo et al., 2012) and potentially undertaking any form of digitalization practices. Rural people are the economic bedrock of many African countries; not only are they the majority in most areas, but their contribution through agricultural activities is central to development (Barrett et al., 2017; Dixon, 2015; Osabuohien, 2020). Meanwhile, the conditions under which rural farmers undertake their livelihood activities and everyday practices are mostly far from ideal. Poor infrastructures such as roads, electricity, and now telecommunication, to lack of social services like education and health, the plights of rural communities and people have always been at the centre of rural development scholarships, as well as development interventions (Barrett et al., 2017; Briceño-Garmendia et al., 2009; Cook, 2011). Notably, the outcome of these many problems is more rural poverty and thwarted abilities to embrace innovations and change entirely. For example, the lack of education in rural areas leads to low literacy, hindering people's access to information, including digital tools and services. Ultimately, the practice(s) of digital agriculture hinges on how rural people are situated to adopt, use, and participate in the phenomenon.

This paper uses a cross-sectional survey in four districts in Northern Ghana to assess the factors that may influence adoption and participation in digital services. By showing the effects of various farmer characteristics on the involvement in digitalization, we provide empirical and practical insights into how the everyday realities of rural people in Africa may influence the goals of the anticipated digital revolution in the region. The rest of this paper is structured as follows. The following section presents background on agricultural digitalization in Africa. Thereafter, the research design and data collection processes are outlined, followed by the empirical approach, where the variables are defined, and the model for the research is stated. The results section then presents the outcome of the probit regression modelling and how each variable influences participation. Then the discussion expands and provides reasons for the five significant determinants (association to farmers groups, extension services, gender, ability to place calls, and access to cellular internet) of participation in digital services. The conclusion and way forward summarise the findings and call for attention to diverse groups of farmers’ access to resources and digital literacies in digital interventions.

## 2. Background literature on agriculture digitalization in smallholder systems

### 2.1. Context of digitalization in smallholder Africa

In 2019, the Food and Agriculture Organisation released *Digital technologies in agriculture and rural areas–status report* (FAO, 2019). The report emphasizes the growing application of many digital tools, including mobile advisories, precision advisories, satellite imagery, blockchains, and drones inb many aspects of rural smallholder food and agriculture value chains. In the same year, the CTA released *The Digitalisation of African Agriculture Report 2018–2019* (Technical Centre for Agricultural and Rural Cooperation, 2019). The report detailed the growing number of digital innovations, such as call centres, market platforms, blockchain solutions, and social media. Two reports released by GIZ and partners in 2021 (Deutsche Gesellschaft für Internationale Zusammenarbeit, Mercy Corps AgriFin, & Dalberg, 2021; Deutsche Gesellschaft für Internationale Zusammenarbeit, Mercy Corps, Mercy Corps AgriFin, & Dalberg, 2021) also emphasized how digital tools and services are becoming embedded in everyday activities of farmers and rural people in Africa. Similar information on the subject can be found in the World Bank (Kim et al., 2020), GSMA Association (GSM Association, 2020), the African Union (African Union Commission & OECD, 2021), and many other organizations. In all these reports, the common theme is the emergence of a new potentially transformative technology and phenomena that are rapidly penetrating through the fibre of rural communities and activities. This view is also heavily supported in the academic literature on digitalization, which engages the subject while acknowledging the growing proliferation of digitalization in African rural agriculture (Babcock, 2015; Ekekwe, 2017; Evans, 2018; Munyua et al., 2008).

According to the CTA, about 33 million smallholders registered for digital services in 2019, and the number is expected to reach 200 million by 2030 (Tsan et al., 2019). However, little is known about the factors affecting farmers’ digital services participation. Researchers have shown that various factors broadly influence people's adoption of ICTs and digitalization in agriculture. Socio-demographic and economic conditions such as education, gender, income, social category, and age variedly affect farmers’ adoption and application of ICTs (Alabi, 2016; Ali, 2012; Ayanwale and Adekunle, 2008; Ayim et al., 2020; Etwire et al., 2017; Tata and McNamara, 2016). Likewise, Ajani (2014) note that literacy and digital skills are critical to scaling digitalization for smallholders in rural Africa. Specifically, illiteracy, financial illiteracy, and digital illiteracy, as Babcock (2015) found with mobile payments, are barriers to successful adoption and use.

The literature on the farmers’ use of digitalization has been skeptical about unequal access and equity among diverse groups (Bronson, 2018; Carolan, 2020; Duncan et al., 2021; Klerkx et al., 2019; Rotz et al., 2019). Hence, there is no denying in the literature that digitalization, despite the potential, may not be available for all farmers (Emeana et al., 2020; Xie et al., 2021), and access and use could be dependent on a diverse range of factors. The concerns on the potential non-inclusiveness of access and benefits of digitalization (Abdulai, 2022a) piqued my interest in further exploring this subject. This paper, therefore, models the effects of a range of factors on participation in digital services within rural Northern Ghana using experiences of common digitalization's in Africa.

### 2.2. Digital agriculture services in Africa

The ubiquity of digitalization and accompanying services lies in the diverse range of tools and services emerging from the phenomenon. These innovations mainly provide solutions to the many challenges smallholders and rural farmers face in the current food regime, including climate change, poor access to inputs, and limited access to information. Digitalization manifests in farmers’ access and use of the digital hardware/tool, software and services for farming activities. It includes direct or indirect use of simple digital devices (e.g., phones, computers, radios, tablets, etc.) and more advanced digital hardware (drone, satellite/GIS, field sensors, machinery sensors, portable soil/crop/ input diagnostics precision systems). Other digital hardware and software (e.g., data capture tools, field agent management tools, data analytics tools, and blockchain platforms) are also important, as well as the use of data (e.g., farmer registries, farmer transactions, soil maps, weather, pest & disease surveillance) to create services for farmers (Babcock, 2015; Deutsche Gesellschaft für Internationale Zusammenarbeit et al., 2021; GSM Association, 2020; Tsan et al., 2019). Hence, from receiving weather alerts on mobile phones to reaching out to call centres to drone use for spreading fertilizers and controlling pests, farmers can use digital-enabled hardware and equipment for various farm tasks, to market access solutions that connect an otherwise disconnected smallholder population to markets and bridge price information asymmetries (Deichmann et al., 2016; Etwire et al., 2017; Magesa et al., 2014), digitalization is extensive.

The Technical Centre for Agricultural and Rural Cooperation (2019) has classified these emerging digital agricultural services into five categories: **1) *Advisory and information services*** that provide farmers with information on diverse topics, including agronomic practices, weather, and market information; 2) ***Market linkages*** platforms to link smallholder farmers to input and output markets; 3) ***Supply chain management services*** that connect different levels of the agri-food supply chain actors in ways that allow for greater efficiency and transparency; 4) ***Financial access services*** that provide digital financial solutions such as payments, savings, and insurance for smallholders; and 5) ***Macro agricultural intelligence services*** that integrate a variety of data sources to provide valuable country and value-chain level insights and decision tools for government policymakers, extension agencies, agronomists, agribusinesses, and investors. These services are offered to smallholders through the digital tools and hardware noted earlier, with mobile phone being the most widely used (GSM Association, 2020; Tsan et al., 2019). Common ways of interactions with farmers include radio programmes, call centres, Interactive voice Response (IVR), SMS messages, and field agents.

## 3. Research design and data collection

**Figure 1.**
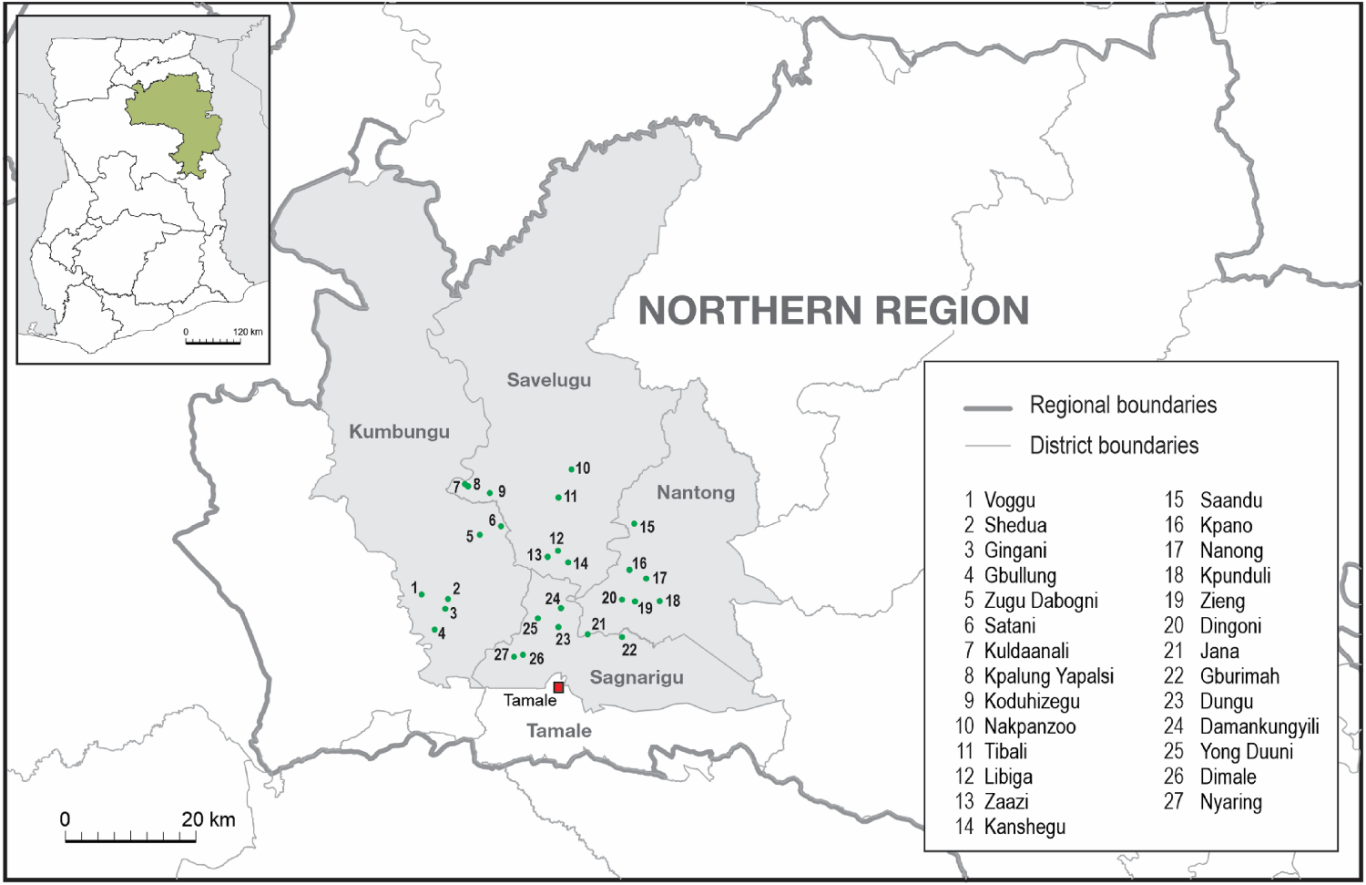
Map of the study area and communities.

This paper uses data from a cross-sectional survey administered in the Northern Region of Ghana in 2021. The study's goal was to assess the perceptions and preparedness of rural farmers for the ongoing digital revolution. A multistage sampling method selected participating smallholder farmers (n = 1565). First, four districts (Savelugu, Nantong, Kumbungu, and Sagnarigu) (see Figure 1) were chosen due to their proximity to the regional capital district, Tamale Metropolis. Their locations make these areas potential beneficiaries of digital services of urban communities yet are primarily peasant-based enough to offer insights into rural digitalization. Their closeness to the regional capital also makes them accessible to the many NGOs that operate from the city and provide agricultural interventions for rural smallholders. These communities presented an opportunity to view people who have or are currently experiencing digitalization and non-beneficiaries in such communities.

The communities (27) were selected through a simple random sampling from a pool of areas that received digital agriculture interventions. Finally, a systematic sampling technique was used to choose household units by selecting every third house where the research team first entered the community/village. Data was specifically collected from members of the selected smallholder households (as broadly defined in initiatives to include all farmers in rural communities) targeting the head since registration for services mainly focused on them. However, in cases the head designated another member as a point person for services, we focused on the that member. In the absence of the head, the next available member in position to speak to the issues were surveyed. This approach worked well since majority of people in the communities were farmers and had opportunities to engage with digital services (see Abdulai, 2022a for further details). The survey covered different areas of digitalization, including household preparedness, perceptions of usage, impacts, challenges, and pathways to sustainable and inclusive digitalization. Ethical approval was granted by the University of Guelph Research Ethics Board.

The data was collected from in-person administered surveys through tablets in a digital format with the help of research assistants. The survey was conducted with a structured questionnaire, capturing farmer characteristics, experiences, and perceptions. The enumerators were trained on the study and the data collection and assigned communities and distributed to sections in the selected areas on the survey days.

## 4. The Empirical Approach

We adopted the following probit model equation following the methods in the literature (KC et al., 2016; Kc et al., 2019)

$$In\frac{\;p}{1-p}=\beta_{o}+\beta i\overset{n}{\underset{i=1}{\sum }}\;\mathrm{Xi}$$

Where p = the probability that a farmer participates in the digitalization of agriculture services, and βo and βi are coefficients. Participation in digitalization was measured through a simple survey question on whether participants had engaged with digitalization services or not. Since the propensity index is not directly observable, it is treated as a latent variable to develop a model that explains the probability a farmer is likely to participate in digital agricultural services (1 = yes, participated in digital service, 0 = no, no participation in digital services) when a change occurs in any of the explanatory variables (Xi) pertaining to the farmers’ socio-economic conditions (gender, age, education, membership in association, and access to extension services), digital competencies (ability to place phone calls, send SMS, follow IVR, or browse the internet), and access to digital tools and resources (phones and internet) (see next section for details).

Ultimately, we apply the model that tried to understand the propensity of a farmer to participate in a digital service offered in their communities by service providers. After multiple modelling iterations, we established a robust model of participation based on 11 variables (Table 1). The 11 variables were concluded based on researchers’ experiences in the field, anticipations of important determinants, preliminary qualitative insights, and elimination of incomplete and redundant variables. Microsoft Excel and R statistics were used for the relevant analysis. Excel was used to compute frequencies and percentages, and R statistics was used for the statistical analysis.

**Table 1.** Summary of key variables in the model.

Farmer characteristics			
Variable (N-1565)		Mean	Standard Deviation
Age		38.88	12.98
Variable	Options	Frequency	Percentage (%)
Gender	Female	617	39.42
	Male	948	60.58
Age	15–24	121	7.73
	25–40	891	56.93
	41–60	440	28.12
	60 +	113	7.22
Level of education	No education	1080	69.01
	Basic education (incomplete)	243	15.53
	Basic education (complete)	106	6.77
	High school	104	6.65
	Certificates/vocational	12	0.77
	Higher education	20	1.28
Membership of association	No	511	32.65
	Yes	1055	67.35
Access to extension services	No	479	30.61
	Yes	1086	69.39
Respondents’ access to digital tools and resources			
Respondents with access to:		Percentage WITHOUT access (%)	Percentage WITH access (%)
Phone		2.17	97.83
Internet		86.96	13.04
Farmers digital competencies			
Respondents who can:		Percentage WITHOUT ability (%)	Percentage WITH ability (%)
place calls		10.35	89.65
send SMS messages		68.75	31.25
follow IVR		79.23	20.77
browse the internet for information		82.75	17.25

## 5. Results and findings

### 5.1. Summary statistics for key variables

Eleven independent variables were considered to assess their relationship with farmers’ participation in digital services.

Most respondents were male (60.48%), while females (29.42%) were the minority. Most respondents were between the ages of 25 and 40 (56.93%), and the average age was 38.8 years. Most farmers had no education (69.01%). However, the majority of farmers belonged to associations (67.35%) and had access to extension services (69.39%). While most farmers owned or had access to mobile phones (97.83), only 13.04% had internet access. The commonest competencies among farmers were the ability to place (89.65%), and the least digital competence was browsing the internet for information (17.77%). Generally, as the complexity or level of skill required to undertake a particular digital task increased, the percentage of farmers who could complete such a task decreased (Table 1). Finally, the dependent variable measured was farmers’ participation in digital agriculture services in the area: 70.22% of the respondents had participated in digital services.

### 5.2. Critical factors associated with farmer participation in digital agricultural services

The probit regression illustrated that a range of factors explained farmers’ participation in digital services. The overall regression model explained participation reasonably well (R^2^ = 0.376). The explanation power was acceptable in line with a cross-sectional dataset like this study (Kc et al., 2019). Table 2 outlines the findings of our general model for participation in digital agricultural services.

- Gender had a significant positive relationship with participation in digital services. Specifically, male farmers are more likely to participate than women. The marginal effect computation indicated that a unit increase in men in farming led to 6.7% increase in participation in digital services.
- Membership of association also had a strong and positive effect on farmers’ participation. Particularly, the calculation of the marginal effect indicated that a unit increase in association to groups among farmers increased participation in digital services by 27%.
- Access to extension services was also strongly associated with farmers participation in digital services. The estimated model coefficient for access to extension services was positive. The marginal effect calculation showed that a unit increase in farmers with access to extension services increased the probability of participating in digital agricultural services by 17%.
- The overall model also expressed the estimated coefficient for access to mobile phones as positive. Compared to farmers without ownership or access to mobile phone, those who had access to phone were 11% more likely to participate in digital services.
- Farmer's ability to place a call had a significantly positive coefficient, and increased farmers participation in digital services. Particularly, farmers who can place phone calls were more likely to participate in digitalization. The marginal effect calculation revealed that farmers a unit increase in farmers who can place phone calls increase the probability of participation in digital services by 15%.

**Table 2.** Determinants of participation in digital services.

Variable n = 1565	General model Coefficient (Standard error)	General model marginal effect
(Intercept)	−1.902 (0.335)	−0.397
Gender *1 if a participant is male; 0 otherwise*	0.322*** (0.089)	0.067
Age *Age of household head (years)*	−0.003 (0.003)	−0.001
Level of education *1 if a farmer ‘has completed at least basic school: 0 otherwise*	0.089 (0.139)	0.018
Membership of association *1 if a farmer is a member of an association; 0 otherwise*	1.318*** (0.100)	0.275
Access to extension *1 if farmer has access to extension; 0 otherwise*	0.820*** (0.102)	0.171
Phone *1 if farmer has a phone; 0 otherwise*	0.528** (0.265)	0.110
Internet access Cellular *1 if farmer has cellular internet; 0 otherwise*	−0.737*** (0.123)	−0.154
I can place calls on my phone *1 if a farmer can place calls; 0 otherwise*	0.747*** (0.130)	0.156
I can send SMS messages *1 if a farmer can send SMS; 0 otherwise*	0.055 (0.133)	0.0115
I can follow IVR on my phone *1 if a farmer can follow IVR; 0 otherwise*	−0.165 (0.198)	−0.034
I can browse the internet for information *1 if farmer can browse the internet; 0 otherwise*	0.139 (0.220)	0.029
Constant	−1.902*** (0.335)	
Log Likelihood	−594.829	
McFadden's Pseudo R-squared	0.376	

Note: *p < 0.1; **p < 0.05; ***p < 0.01.

Age, level of education, ability to send SMS, follow an IVR, and browse the internet did not significantly affect farmers’ participation in digital services. However, each of these elements (except ability to follow IVR) had positive coefficients and increased farmers participation in digital services.

In all, gender, membership in farm associations, access to extension services, ownership and access to mobile phones, access to cellular internet, and ability to place calls largely affected participation in digital services in Northern Ghana (Table 2). However, these results represent an exploration of the variables at the time of the research, which is subject to changes in the future as digitalization expands to cover new form of technologies and services. Hence, care is needed in interpreting the results due to potential changes that may emanate from changing circumstances and requirements of digital services in the area. The results must be considered an exploration highlighting some key elements influencing digitalization, but not as emphatic determinants.

## 6. Discussion

### 6.1. Key factors that determines smallholder participation in digital services

Rural smallholder farming systems in Africa are increasingly exposed to the digitalization of agriculture through various digital services, but our results show that access may not be uniform. About 33 million smallholders registered for digital services at the end of 2019, and the number is expected to reach 200 million by 2030 (Tsan et al., 2019). However, as Abdulai (2022) has argued, existing and historical structures, for example, class dynamics and access to resources, may lead to inequities in rural areas. The analysis in this paper has shown five key factors that could influence access and participation in digital services.

The most significant factor that affected participation in digital services was farmers association to associations. This result is unsurprising because the provision of development interventions through groups is a well-known development strategy in Africa (Ainembabazi et al., 2017; Baah, 2008; Mwaura, 2014). And since digitalization interventions are primarily situated within the international development activities of NGOs (Abdulai, 2022), the adoption process mimics prior development experiences. Hence, farmers are sometimes put into groups as a prerequisite for receiving digital services. These elements partly explain why people associated with groups are likely to participate. Importantly, this finding confirms earlier research that farmer groups and cooperatives improve the adoption of innovations and technologies in agriculture (Abegunde et al., 2020; Ayanwale and Adekunle, 2008; Sulo et al., 2012; Wossen et al., 2017). Furthermore, associations to groups are known to speed up adoption processes. In the study of the adoption of technologies in African grate lake regions, Ainembabazi et al. (2017) found that farmer groups are not just valuable for promoting adoption, but they also reduce adoption lag in smallholder systems. This paper partly confirms this assertion since much of the participation in Northern Ghana is early adoption of digital services. Hence, attention must be paid to local farmer grouping in rural areas in the face of emerging technologies.

The second most important variable in farmers participation in digital services was access to extension services. Particularly, we found that access to extension increased the probability for farmers to join digital services. This finding confirms the critical role agricultural extension in transfer of innovations. An extensive body of literature have discussed how extension services is by government and private entities to introduce and facilitate technology and innovation diffusion in agriculture (Eastwood et al., 2017; Ruttan, 1996; Sodiya et al., 2008). Particularly, researchers have shown that extension is critical in adoption and use of technologies in agriculture (Ali and Rahut, 2013; Emmanuel et al., 2016; Tata and McNamara, 2016; Wossen et al., 2017) by increasing farmers access to information and innovations. We have, therefore, empirically added to this claim and extended the literature by showing that extension is also essential in the diffusion and use of digital agricultural technologies.

The third and fourth important factors in our model that affects participation was farmers’ digital competence in placing phone calls and their access to mobile phones respectively. Particularly, access to mobile phones increased the probability of participation in services and the ability to use them. These findings were anticipated due to the current space of digitalization in Africa and Ghana, where majority of digital agricultural services are created and delivered through mobile phones (Babcock, 2015; Emeana et al., 2020; GSM Association, 2020; Tsan et al., 2019). Notably, most digital services are mobile based, either through SMS advisories or call centres, both of which use the digital competency of operating a phone Emeana et al. (2020) has described digitalization as a revolution of mobile-enabled services. Hence, mobile penetration (Duncombe, 2016; GSMA Association, 2021) and ability to use these tools (Abdulai, 2022b; McCampbell et al., 2021; Tsan et al., 2019) are critical for scaling of digital services. Importantly, the findings underscore the essence of digital tools and competencies in promoting smallholder digitalization. The critical role of mobile phones mean there are prospects for inclusive scaling of digital services as phones are widespread in Africa. For example, mobile subscriptions in Ghana exceeds 100%, which shows a high usage in the country (2020 – GSMA Mobile Connectivity Index, n.d.). The ubiquity of mobile phones lends itself to growing opportunities for farmers to engage with digital tools and develop competencies to use them to benefit from digital services and solutions. However, as Ajani (2014) noted, when it comes to smallholders in rural Africa, issues of low literacy and digital skills continue to undermine adoption and use. Atanga (2020) confirms the challenge of literacy when he found that low technical ability among farmers hinders digital financial services’ ability to make impacts for smallholders in Ghana. Establishing the essence of digital skills (placing phone calls in this case) opens further conversations on developing digital competencies in rural areas.

The fifth variable that significantly affects participation in digital services was gender. Gender has long been a critical dimension in technology and innovation adoptions in Africa, due in part to socio-cultural set-ups in the area (Doss, 2001; Lambrecht et al., 2018). Estimates show that there is between 4%-40% difference in agricultural productivity between male and female farmers in Sub-Sahara Africa, favouring men (Kilic et al., 2015) due in part to differential access to productive resources, including land and agricultural inputs (such as technologies). Specific to the digital innovation space, women are still 13% less likely to own a mobile phone than men in sub-Saharan Africa- which is a crucial tool required for digital participation in the current ecosystem (GSMA Association, 2021). This study thus confirms this unequal access to resources as male farmers are more likely to participate in digital services. Another reason for a difference could also result from emphasis on development interventions at the household level, which places men as household heads at the advantage of participation. However, recent studies have revealed the gradual blurring of such gendered barriers. In the study of agrarian transformation, Speijer (2016) found transformations in access to inputs, land, technology and labour at gender levels. Long-held beliefs of division between male and female crops are blurred as technologies alter access to horsepower for farm activities, confirming Doss (2001) and Lambrecht et al. (2018) findings on disappearing gender myths on limited access to resources by women in Africa. However, as this study partly reveals, there is still room for improvements despite the blurring of gender constraints. Hence, interventions that promote and offer digital services in Africa must continuously integrate gender-sensitive assessment in planning and implementation processes.

### 6.2. Digitalization as an uneven phenomenon for smallholder participation?

The results show variations in the probability and likelihood of participating in agricultural digitalization services. Specifically, different characteristics of farmers engender different effects on farmers’ interest and desire to use available services. The revelation of how these elements variedly affect the likelihood of participation speaks to issues of potential uneven access and benefits in digitalization. As noted, 69.01% of the rural population had no formal education; 86.96% had no internet access; 68.75% and 79.23% could not send a simple SMS or follow IVR, respectively. Likewise, 10.35% could not place a phone call, and 2.17% did not own or have access to a mobile phone. Meanwhile, the model shows that these factors influence the probability of participation and engagements with digital services. Hence, groups such as women, digital illiterates, people without access to extension services, and those not affiliated with farm associations risk being excluded from participation and potential benefits.

The findings confirm and empirically support claims that digitalization may be unevenly accessible and inclusive to all farmers, as well as other researchers who have questioned how digitalization may lend itself to uneven access (Carolan, 2018, 2020; Rose and Chilvers, 2018; Rotz et al., 2019) similar to older technologies and innovations in the earlier green revolution efforts (Abdulai, 2022c). For example, illiterates are less likely to adopt and participate in services without adequate measures to help them understand messages delivered in digital initiatives. These inequities partly explain why digitalization have yet to take hold among smallholders (Tsan et al., 2019). Such disparities may continue to undermine any potential for full-scale and inclusive digitalization of agriculture. And could further deepen existing inequalities among rural farmers (including gender and literacy disparities) while also creating newer classes along the lines of groups with advantages of access (Abdulai, 2022c). In essence, digitalization is essentially no different from other technological innovations criticized for creating, entrenching, and thriving on existing and newer socio-economic inequities unless conscious efforts are instituted for inclusive scaling.

### 6.3. Some policy directions for inclusive digital services participation

The model shows differential effects of varied farmers’ characteristics on the scaling of digitalization among rural farmers and smallholders. Hence, we draw normative policy directions from the marginal impacts of the model to make recommendations for inclusiveness and widespread participation in digital agricultural services.

The first policy recommendation is promoting and growing farmer participation in farm/community associations and groups. The marginal effect indicated that a unit increase in association with farmer groups would increase the likelihood of involvement in digital services by 27%. Thus, there would be a corresponding increase in participation in digital services for any rise in the number of people participating in farm groups. Meanwhile, only 67.35% of the sampled population participated in farmer organizations. Hence, measures must be instituted by public and private actors to generate interest and grow the proportion of farmers who participate in associations or groups in their communities. These measures could range from creating group-based models for service provisions to incentivising the formation of farmer associations.

The second key policy direction is increasing farmer access to extension services. As earlier noted*,* 30.61% of the farmer population had no access to extension services. Meanwhile, the model showed that a unit increase in access to an extension would increase the probability of participation in digital agricultural services by 17%. Thus, pursuing inclusive access to extension services through strategies that make extension agents available, and farmers motivated in using them could increase participation. Hence, governments must institute policy activities that enhance farmer extension access to improve the inclusiveness of digital innovations in smallholder systems. Likewise, creating avenues for private extension programme could increase access in ways that enhance farmers knowledge of innovations such as digitalization services.

The third policy recommendation is growing farmers’ access and ownership of mobile phones, as well as increasing their competencies in usage. Thus, prioritizing inclusive access to mobile phones and digital competencies would increase people's interest in experiencing various digital services. This policy direction is critical as current digitalization efforts primarily rely on mobile phones, which makes it an indispensable prerequisite to scaling digitalization in smallholder systems. Importantly, access without competence cannot be regarded as complete as knowledge of usage is needed for farmers to fully utilize mobile phones. Hence, measures such as farmer training programmes, informal education schemes, or farmer-to-farmer education that focuses on digital literacies must be instituted in rural communities to train farmers in various digital competencies required for effective adoption and participation in digitalization.

## 7. Summary, conclusion and limitations

The paper has explored the factors that influence the likelihood of rural smallholder participation in digital agricultural services. The results showed that gender, membership in farm associations, access to extension services, ownership and access to mobile phones, and ability to place calls primarily increased the likelihood of participation in digital services in Northern Ghana. Age, level of education, ability to send SMS, follow an IVR, and browse the internet did not significantly affect farmers’ participation in digital services. However, these elements (except the ability to follow IVR) had positive coefficients and increased farmers’ involvement in digital services. The findings lend critical policy insights for inclusive agriculture digitalization in smallholder systems. A place-based understanding of factors that drive participation, penetration and scaling of digital services is essential. Development stakeholders must pay attention to the socio-political-economic conditions of smallholders and rural farmers when designing and implementing digital initiatives. Specifically, strategies must be pursued to increase farmers’ access to digital resources (e.g., phones, internet, etc.). Likewise, attention must be placed on digital literacies among farmers. Governments, NGOs, and all rural development actors need to institute and prioritize education and training programmes that enhance the competencies of rural people. Also, measures that increase access to and encourage extension services are needed. Finally, farmer associations and groups must be encouraged in rural areas, and incentives created to promote participation and belonging to such groupings. Ultimately, through these policy directions and strategies, governments and development agencies would have effectively empowered diverse rural people to participate in digital services and propel inclusive digital futures.

Finally, the study has some limitations despite the critical contributions. The modelling has largely focused on individual socio-economic characterises that influence farmers engagement with digitalization services. These results represent an exploration of the variables at the time of the research. Hence, the significant elements must not be considered exclusive or exhaustive; because other factors such as farmer motivations and values and structural institutional and political economic conditions shown to influence farmers behaviours could exert unaccounted impacts (Abdulai, 2022b, 2022c; Quarshie et al., 2021; Ruttan, 1996). For example, most digital services are currently a part of development interventions, which could ultimately define who can potentially access, participate and benefit from them. Hence, care is needed in interpreting the results due to changing circumstances and requirements of digital services in the study settings. Further research must explore other dimensions of participation in diverse contexts and across times.

## References

[bibr1-00307270221144641] *2020 – GSMA Mobile Connectivity Index* (n.d.) 2020 – GSMA Mobile Connectivity Index. Retrieved 31 March 2022, from https://www.mobileconnectivityindex.com/.

[bibr2-00307270221144641] Abdulai A-R (2022a) *The Digitalization of Agriculture and the (Un)Changing Dynamics of Rural Smallholder Farming Systems in Ghana, Sub-Sahara Africa* [PHD, University of Guelph]. https://hdl.handle.net/10214/27141.

[bibr3-00307270221144641] Abdulai A-R (2022c) A new green revolution (GR) or neoliberal entrenchment in Agri-food systems? Exploring narratives around digital agriculture (DA), food systems, and development in sub-sahara Africa. The Journal of Development Studies 0(0): 1–17.

[bibr4-00307270221144641] Abdulai A-R (2022b) Toward digitalization futures in smallholder farming systems in Sub-Sahara Africa: A social practice proposal. Frontiers in Sustainable Food Systems 6, 10.3389/fsufs.2022.866331.

[bibr5-00307270221144641] Abegunde VO Sibanda M Obi A (2020) Determinants of the adoption of climate-smart agricultural practices by small-scale farming households in king cetshwayo district municipality, South Africa. Sustainability 12(1): 95.

[bibr6-00307270221144641] African Union Commission & OECD (2021) Africa’s Development Dynamics 2020: Digital Transformation for Quality Jobs. OECD. 10.1787/0a5c9314-en.

[bibr7-00307270221144641] Ainembabazi JH van Asten P Vanlauwe B , et al. (2017) Improving the speed of adoption of agricultural technologies and farm performance through farmer groups: Evidence from the great lakes region of Africa. Agricultural Economics 48(2): 241–259.

[bibr8-00307270221144641] Ajani EN (2014) Promoting the use of information and communication technologies (ICTs) for agricultural transformation in sub-saharan Africa: Implications for policy. Journal of Agricultural & Food Information 15(1): 42–53.

[bibr9-00307270221144641] Alabi O (2016) Adoption of information and communication technologies (ICTs) by agricultural science and extension teachers in Abuja, Nigeria. Journal of Agricultural Education 57(1): 137–149.

[bibr10-00307270221144641] Ali A Rahut DB (Eds) (2013) Impact of agricultural extension services on technology adoption and crops yield: Empirical evidence from Pakistan. Asian Journal of Agriculture and Rural Development. 10.22004/ag.econ.198306.

[bibr11-00307270221144641] Ali J (2012) Factors affecting the adoption of information and communication technologies (ICTs) for farming decisions. Journal of Agricultural & Food Information 13(1): 78–96.

[bibr12-00307270221144641] Atanga SN (2020) *Digitalization of Agriculture: How Digital Technology is Transforming Small-Scale Farming in Ghana* [Masters]. International Institute of Social Studies.

[bibr13-00307270221144641] Ayanwale AB Adekunle A (2008) Factors determining ICT adoption in rural smallholder farms in southwestern Nigeria. Journal of Social Development in Africa 23(2), Article 2. https://doi.org/10/fn6pdg.

[bibr14-00307270221144641] Ayim C Kassahun A Tekinerdogan B , et al. (2020) Adoption of ICT innovations in the agriculture sector in Africa: A Systematic Literature Review. *ArXiv Preprint ArXiv:2006.13831*.

[bibr15-00307270221144641] Baah F (2008) Harnessing farmer associations as channels for enhanced management of cocoa holdings in Ghana. Scientific Research and Essays 3(9): 395–400.

[bibr16-00307270221144641] Babcock LH (2015) *Mobile Payments: How Digital Finance is Transforming Agriculture*. 65.

[bibr17-00307270221144641] Barrett CB Christiaensen L Sheahan M , et al. (2017) On the structural transformation of rural Africa. Journal of African Economies 26(suppl_1): i11–i35.

[bibr18-00307270221144641] Briceño-Garmendia C Smits K Foster V (2009) *Financing Public Infrastructure in Sub-Saharan Africa* .

[bibr19-00307270221144641] Bronson K (2018) Smart farming: Including rights holders for responsible agricultural innovation. Technology Innovation Management Review 8(2): 7–14.

[bibr20-00307270221144641] Carolan M (2018) ‘Smart’ farming techniques as political ontology: Access, sovereignty and the performance of neoliberal and not-so-neoliberal worlds. Sociologia Ruralis 58(4): 745–764.

[bibr21-00307270221144641] Carolan M (2020) Automated agrifood futures: Robotics, labor and the distributive politics of digital agriculture. The Journal of Peasant Studies 47(1): 184–207.

[bibr22-00307270221144641] Cook P (2011) Infrastructure, rural electrification and development. Energy for Sustainable Development 15(3): 304–313.

[bibr23-00307270221144641] Deichmann U Goyal A Mishra D (2016) Will digital technologies transform agriculture in developing countries. Agricultural Economics 47(S1): 21–33.

[bibr24-00307270221144641] Deutsche Gesellschaft für Internationale Zusammenarbeit, Mercy Corps AgriFin, & Dalberg (2021) *Digital Agriculture Platforms Bleuprints-White Paper*. GIZ.

[bibr25-00307270221144641] Deutsche Gesellschaft für Internationale Zusammenarbeit, Mercy Corps, Mercy Corps AgriFin, & Dalberg (2021) *Digital Agriculture Platforms. Blueprints Deep-Dive Report*. GIZ. https://www.mercycorpsagrifin.org/wpcontent/uploads/2021/01/GIZ_Dalberg_Blueprint_Intro-Blog-2021.pdf

[bibr26-00307270221144641] Dixon C (2015) Rural Development in the Third World. Routledge. 10.4324/9781315685755.

[bibr27-00307270221144641] Doss C (2001) How does gender affect the adoption of agricultural innovations? The case of improved maize technology in Ghana. Agricultural Economics 25(1): 27–39.

[bibr28-00307270221144641] Duncan E Abdulai A-R Fraser EDG (2021) Modernizing agriculture through digital technologies: Prospects and challenges. *Handbook on the Human Impact of Agriculture*. https://www.elgaronline.com/view/edcoll/9781839101731/9781839101731.00018.xml.

[bibr29-00307270221144641] Duncombe R (2016) Mobile phones for agricultural and rural development: A literature review and suggestions for future research. The European Journal of Development Research 28(2): 213–235.

[bibr30-00307270221144641] Eastwood C Klerkx L Nettle R (2017) Dynamics and distribution of public and private research and extension roles for technological innovation and diffusion: Case studies of the implementation and adaptation of precision farming technologies. Journal of Rural Studies 49: 1–12.

[bibr31-00307270221144641] Ekekwe N (2017, May 18) *How Digital Technology Is Changing Farming in Africa*. Harvard Business Review. https://hbr.org/2017/05/how-digital-technology-is-changing-farming-in-africa.

[bibr32-00307270221144641] Emeana EM Trenchard L Dehnen-Schmutz K (2020) The revolution of Mobile phone-enabled services for agricultural development (m-Agri services) in Africa: The challenges for sustainability. Sustainability 12(2): 85.

[bibr33-00307270221144641] Emmanuel D Owusu-Sekyere E Owusu V , et al. (2016) Impact of agricultural extension service on adoption of chemical fertilizer: Implications for rice productivity and development in Ghana. NJAS - Wageningen Journal of Life Sciences 79: 41–49.

[bibr34-00307270221144641] Etwire PM Buah S Ouédraogo M , et al. (2017) An assessment of mobile phone-based dissemination of weather and market information in the Upper West Region of Ghana. Agriculture & Food Security 6(1), https://doi.org/10/ghff2b.

[bibr35-00307270221144641] Evans O (2018) Digital agriculture: Mobile phones, internet and agricultural development in Africa. Actual Problems of Economics 7-8: 76–90.

[bibr36-00307270221144641] FAO (2019) *Digital technologies in agriculture and rural areas—Status report*. 152. http://www.fao.org/3/ca4985en/ca4985en.pdf.

[bibr37-00307270221144641] GSMA Association (2021) *GSMA – Connected Women – The Mobile Gender Gap Report* 2021 (p. 63). GSM Association.

[bibr38-00307270221144641] GSM Association (2020) *The mobile economy Sub-Saharan Africa 2020*. GSM Association. https://www.gsma.com/mobileeconomy/wp-content/uploads/2020/09/GSMA_MobileEconomy2020_SSA_Eng.pdf.

[bibr39-00307270221144641] KC KB Pant LP Fraser EDG , et al. (2016) Assessing links between crop diversity and food self-sufficiency in three agroecological regions of Nepal. Regional Environmental Change 16(5): 1239–1251.

[bibr40-00307270221144641] Kc KB Seng R Fraser E (2019) Should I stay or should I go? Fishers’ ability and willingness to adapt to environmental change in Cambodia’s tonle sap lake. Fisheries Management and Ecology 26(3): 211–223.

[bibr41-00307270221144641] Kilic T Winters P Carletto C (2015) Gender and agriculture in sub–saharan Africa: Introduction to the special issue. In: Agricultural Economics. Wiley Online Library, Vol. 46, Issue 3, pp.281–284.

[bibr42-00307270221144641] Kim J Shah P Gaskell JC , et al. (2020) Scaling Up Disruptive Agricultural Technologies in Africa. The World Bank. 10.1596/978-1-4648-1522-5.

[bibr43-00307270221144641] Klerkx L Jakku E Labarthe P (2019) A review of social science on digital agriculture, smart farming and agriculture 4.0: New contributions and a future research agenda. NJAS - Wageningen Journal of Life Sciences 90–91: 100315.

[bibr44-00307270221144641] Lambrecht I Schuster M Samwini SA , et al. (2018) Changing gender roles in agriculture? *Evidence from* 20, years of data in Ghana. Agricultural Economics, 49(6), 691–710.

[bibr45-00307270221144641] Magesa MM Michael K Ko J (2014) Access to agricultural market information by rural farmers in Tanzania. International Journal of Information and Communication Technology Research 4(7): 264–273.

[bibr46-00307270221144641] McCampbell M Adewopo J Klerkx L , et al. (2021) Are farmers ready to use phone-based digital tools for agronomic advice? Ex-ante user readiness assessment using the case of Rwandan banana farmers. The Journal of Agricultural Education and Extension 0(0): 1–23.

[bibr47-00307270221144641] Munyua H Adera E Jensen M (2008) Emerging ICTs and their potential in revitalizing small scale agriculture in Africa. *IAALD AFITA WCCA2008*, 11.

[bibr48-00307270221144641] Mwaura F (2014) Effect of farmer group membership on agricultural technology adoption and crop productivity in Uganda. African Crop Science Journal 22: 917–927.

[bibr49-00307270221144641] Osabuohien E (2020) The palgrave handbook of agricultural and rural development in Africa. Palgrave Macmillan 10: 978–973.

[bibr50-00307270221144641] Quarshie PT Abdulai A-R Fraser EDG (2021) Africa’s “Seed” Revolution and Value Chain Constraints to Early Generation Seeds Commercialization and Adoption in Ghana. Frontiers in Sustainable Food Systems 5, 10.3389/fsufs.2021.665297.

[bibr51-00307270221144641] Rose DC Chilvers J (2018) Agriculture 4.0: Broadening Responsible Innovation in an Era of Smart Farming. Frontiers in Sustainable Food Systems 2, https://doi.org/10/ggt3wr.

[bibr52-00307270221144641] Rotz S Gravely E Mosby I , et al. (2019) Automated pastures and the digital divide: How agricultural technologies are shaping labour and rural communities. Journal of Rural Studies. https://doi.org/10/ggkjzk.

[bibr53-00307270221144641] Ruttan VW (1996) What happened to technology adoption-diffusion research? Sociologia Ruralis 36(1): 51–73.

[bibr54-00307270221144641] Sodiya CI Lawal-Adebowale OA Fabusoro E (2008) Effect of Private and Public Extension Services on Adoption of Promoted Cassava-Based Technologies in Ogun State, Nigeria. Journal of Agricultural & Food Information. 10.1300/J108v08n01_05.

[bibr55-00307270221144641] Speijer T (2016) *Agrarian transformations in Ghana: Exploring changes at the level of gender relations*. 33.

[bibr56-00307270221144641] Sulo T Koech P Chumo C , et al. (2012) Socioeconomic factors affecting the adoption of improved agricultural technologies among women in marakwet county Kenya. Journal of Emerging Trends in Economics and Management Sciences 3(4): 312–317.

[bibr57-00307270221144641] Tata J McNamara P (2016) Social factors that influence use of ICT in agricultural extension in Southern Africa. Agriculture 6(2): 15.

[bibr58-00307270221144641] Technical Centre for Agricultural and Rural Cooperation (2019) *THE DIGITALISATION OF AFRICAN AGRICULTURE REPORT* 2018*–*2019. CTA.

[bibr59-00307270221144641] Tsan M Totapally S Hailu M , et al. (2019) *The digitalisation of African agriculture report 2018-2019: Executive summary*. CTA/Dalberg Advisers. https://cgspace.cgiar.org/bitstream/handle/10568/103198/Executive%20Summary%20V4.5%20ONLINE.pdf?sequence = 1&isAllowed = y.

[bibr60-00307270221144641] Wossen T Abdoulaye T Alene A , et al. (2017) Impacts of extension access and cooperative membership on technology adoption and household welfare. Journal of Rural Studies 54: 223–233. 28989229 10.1016/j.jrurstud.2017.06.022 PMC5614096

[bibr61-00307270221144641] Xie L Luo B Zhong W (2021) How Are Smallholder Farmers Involved in Digital Agriculture in Developing Countries: A Case Study from China. Land 10(3), Article 3. https://doi.org/10/gnc9ft.

